# Difference in Uptake of Tetrodotoxin and Saxitoxins into Liver Tissue Slices among Pufferfish, Boxfish and Porcupinefish

**DOI:** 10.3390/md16010017

**Published:** 2018-01-08

**Authors:** Yuji Nagashima, Akira Ohta, Xianzhe Yin, Shoichiro Ishizaki, Takuya Matsumoto, Hiroyuki Doi, Toshiaki Ishibashi

**Affiliations:** 1 Department of Food Science and Technology, Tokyo University of Marine Science and Technology, Minato, Tokyo 108-8477, Japan; crystal.bigfield@gmail.com (A.O.); xianzhe-yin@hotmail.com (X.Y.); ishizak@kaiyodai.ac.jp (S.I.); 2 Department of Environmental Sciences, Faculty of Life and Environmental Science, Prefectural University of Hiroshima, Shobara, Hiroshima 727-0023, Japan; takuya62@pu-hiroshima.ac.jp; 3 Shimonoseki Marine Science Museum “Kaikyokan”, Shimonoseki, Yamaguchi 750-0036, Japan; doi@kaiyukan.com (H.D.); ishibashi@kaikyokan.com (T.I.); 4 Osaka Aquarium Kaiyukan NIFREL, Suita, Osaka 565-0826, Japan

**Keywords:** pufferfish, boxfish, porcupinefish, tetrodotoxin, saxitoxins, liver tissue slice, in vitro incubation, paralytic shellfish toxins

## Abstract

Although pufferfish of the family Tetraodontidae contain high levels of tetrodotoxin (TTX) mainly in the liver, some species of pufferfish, boxfish of the family Ostraciidae, and porcupinefish of the family Diodontidae do not. To clarify the mechanisms, uptake of TTX and saxitoxins (STXs) into liver tissue slices of pufferfish, boxfish and porcupinefish was examined. Liver tissue slices of the pufferfish (toxic species *Takifugu rubripes* and non-toxic species *Lagocephalus spadiceus*, *L. cheesemanii* and *Sphoeroides pachygaster*) incubated with 50 µM TTX accumulated TTX (0.99–1.55 µg TTX/mg protein) after 8 h, regardless of the toxicity of the species. In contrast, in liver tissue slices of boxfish (*Ostracion immaculatus*) and porcupinefish (*Diodon holocanthus*, *D. liturosus*, *D. hystrix* and *Chilomycterus reticulatus*), TTX content did not increase with incubation time, and was about 0.1 µg TTX/mg protein. When liver tissue slices were incubated with 50 µM STXs for 8 h, the STXs content was <0.1 µg STXs/mg protein, irrespective of the fish species. These findings indicate that, like the toxic species of pufferfish *T. rubripes*, non-toxic species such as *L. spadiceus*, *L. cheesemanii* and *S. pachygaster*, potentially take up TTX into the liver, while non-toxic boxfish and porcupinefish do not take up either TTX or STXs.

## 1. Introduction

Pufferfish, belonging to the family Tetraodontidae, accumulate high levels of the potent neurotoxin tetrodotoxin (TTX) and store mainly in the liver and ovary, via the food chain [1,2]. Feeding experiments using *Takifugu niphobles* (at present *T. alboplumbeus*) [3] and *Takifugu rubripes* provide clear evidence that pufferfish accumulate TTX in some organs such as liver, ovary and skin when reared with TTX-containing diets [4,5,6,7,8]. On the contrary, no *T. rubripes* specimens exhibit toxicity when cultured with TTX-free diets in net cages at sea or aquaria on land [9]. It is unlikely that non-toxic species of fish are intoxicated by TTX, because the spotted parrotfish *Oplegnathus punctatus*, Japanese parrotfish *O. fasciatus* and greenfish *Girella punctata* do not accumulate TTX even after culturing with TTX-containing diets [10].

Some species of pufferfish are also reported to contain paralytic shellfish toxins (PSTs). Marine pufferfish of the genus *Arothron* contain considerable amounts of PSTs along with TTX [11,12]. Moreover, in freshwater pufferfish of the genus *Tetraodon* (at present the genus *Leiodon* or *Pao*), including *Leiodon cutcutia*, *Pao fangi*, *P. leiurus*, *P. suvattii* and *P. turgidus* and the genus *Sphoeroides*, PSTs are a major toxin and enough accumulated to cause food poisoning incidents [13,14,15,16]. Pufferfish of the genus *Takifugu* along Japanese coasts, however, do not contain large amounts of PSTs in the liver, although Kodama et al. [17] and Jang and Yotsu-Yamashita [18] detected a trace amount of PSTs in the liver of *T. pardalis* collected from the Northern district of Japan. 

In vitro incubation experiments using liver tissue slices strongly support the results of feeding experiments [9]. In liver tissue slices of *T. rubripes* and *T. pardalis*, accumulation of TTX increases over time when the liver tissue slices were incubated with TTX-containing medium, while in liver tissue slices of non-toxic species of fishes (Japanese parrotfish *O. fasciatus*, greenling *Hexagrammos otakii* and filefish *Thamnaconus modestus*) no increase of TTX-accumulation was observed [19]. Furthermore, *T. rubripes* liver tissue slices incubated in vitro exhibit differences in the accumulation of TTX and PSTs [20]. When *T. rubripes* liver slices were incubated with a medium containing 130 µM TTX and 130 µM PSTs, respectively, the toxin contents were 55 µg TTX/g and 5 µg PSTs/g after 48 h. These findings indicate that *T. rubripes* liver specifically accumulates TTX preferentially over PSTs. Based on the in vitro incubation experiment, moreover, the involvement of carrier-mediated transporter in TTX uptake into liver tissue slices of *T. rubripes* is suggested [21]. Therefore, in vitro incubation experiments are useful and suitable for evaluating the accumulation of toxins in liver among different types of fish.

Although pufferfish of the family Tetraodontidae generally contain TTX, some species of pufferfish such as *Lagocephalus spadiceus* (formerly *L. wheeleri*), *Lagocephalus cheesemanii* (formerly *L. gloveri*) [22], *Lagocephalus lagocephalus* and *Sphoeroides pachygaster* along Japanese coasts are considered non-toxic species according to intensive toxicity tests [23]. In addition, boxfish of the family Ostraciidae and porcupinefish of the family Diodontidae are also non-toxic species [24,25]. In the present study, we investigated TTX and PST uptake into the liver of so-called “non-toxic species” of pufferfish, boxfish and porcupinefish by in vitro incubation experiments using liver tissue slices. TTX was exclusively taken up into the liver tissue slices of pufferfish, including *L. spadiceus*, *L. cheesemanii* and *S. pachygaster* over time with incubation, but not into the liver tissue slices of boxfish or porcupinefish. The PST content in the liver tissue slices of pufferfish was higher than that in boxfish and porcupinefish. The PST content was remarkably lower than the TTX content, however, and did not increase over time with incubation, indicating that the liver of the fish examined in this study did not actively take up PSTs, irrespective of the fish species.

## 2. Results

### 2.1. Uptake of TTX into Liver Tissue Slices of Pufferfish, Boxfish and Porcupinefish

**Figure 1:**
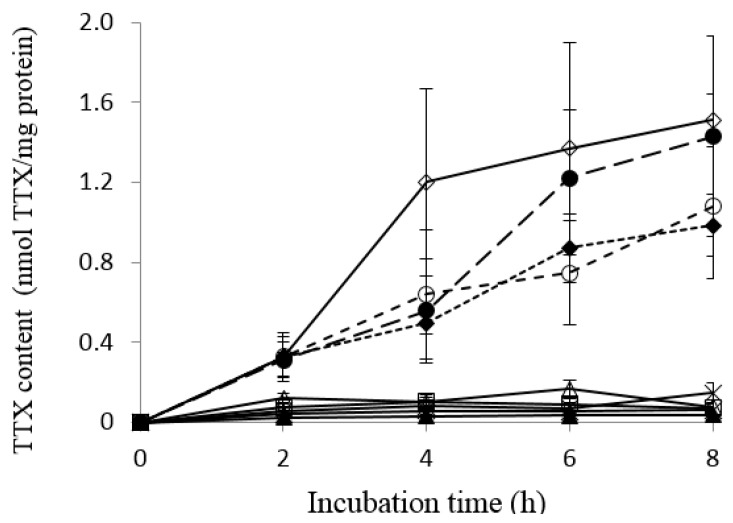
TTX uptake into liver tissue slices of pufferfish, boxfish and porcupinefish. ◇ *Takifugu rubripes*; ● *Lagocephalus cheesemanii*; 〇 *Lagocephalus spadiceus*; ♦ *Sphoeroides pachygaster*; □ *Ostracion immaculatus*; ∆ *Diodon holocanthus*; ▲ *Diodon liturosus*; × *Diodon hystrix*; * *Chilomycterus reticulatus*.

Liver tissue slices of four species of pufferfish (*T. rubripes*, *L. spadiceus*, *L. cheesemanii* and *S. pachygaster*), one species of boxfish (*O. immaculatus*) and four species of porcupinefish (*D. holocanthus*, *D. liturosus*, *D. hystrix* and *C. reticulatus*) were incubated with transport buffer containing 50 µM TTX at 20 °C for up to 8 h. The time-course of TTX uptake into the liver tissue slices is illustrated in Figure 1. None of the liver tissue in the present experiments contained a detectable amount of TTX (<0.3 µg/g liver) or PSTs (<0.4 µg/g liver) before incubation. In tiger pufferfish *T. rubripes* liver tissue slices, a TTX content of 0.336 ± 0.122 nmol/mg protein was detected after 2-h incubation and increased with an increase in the incubation time to 1.51 ± 0.13 nmol/mg protein at 8 h. Similarly, liver tissue slices of the other three species of pufferfish (*L. spadiceus*, *L. cheesemanii* and *S. pachygaster*) also showed increased TTX accumulation over time with incubation. The TTX content at 2 h in *L. spadiceus*, *L. cheesemanii* and *S. pachygaster* was 0.319 ± 0.091, 0.329 ± 0.097 and 0.329 ± 0.019 nmol/mg protein, respectively. At 8 h, the TTX content in liver tissue slices from the three species increased to 1.08 ± 0.36, 1.43 ± 0.50 and 0.985 ± 0.154 nmol/mg protein, respectively, in *L. spadiceus*, *L. cheesemanii* and *S. pachygaster*. 

In contrast, liver tissue slices of boxfish *O. immaculatus* and four species of porcupinefish *D. holocanthus*, *D. liturosus*, *D. hystrix* and *C. reticulatus* exhibited no significant increase in the TTX content over time (*p* > 0.05), although some TTX (0.026–0.166 nmol TTX/mg protein) was detected after 2–8 h incubation. 

### 2.2. Effect of TTX Concentration on TTX Uptake into Liver Tissue Slices of L. spadiceus

**Figure 2:**
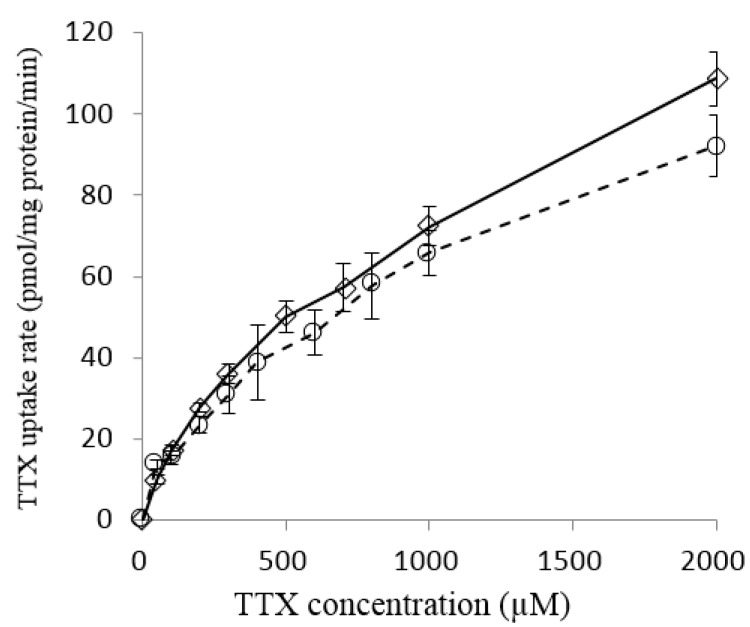
Effect of TTX concentration in the medium on the TTX uptake rate in pufferfish liver tissue slices. 〇 *Lagocephalus spadiceus*; ◇ *Takifugu rubripes*.

Non-toxic species of pufferfish also took up TTX in the liver by the in vitro incubation experiment. Therefore, we examined the effect of the TTX concentration in the transport buffer on the TTX uptake rate into the liver tissue slices of *L. spadiceus* with TTX concentrations ranging from 0 to 2000 µM for 60 min, according to the previously reported method [21]. The uptake rate was dependent on the TTX concentration in the buffer and exhibited a non-linear curve (Figure 2). The profile of the TTX content coincided well with that of *T. rubripes* reported by Matsumoto et al. [21], suggesting that the mechanism of TTX uptake of *L. spadiceus* liver is involved in carrier-mediated transport system, similar to that of *T. rubripes* liver [21].

### 2.3. Uptake of PSTs into Liver Tissue Slices of Pufferfish, Boxfish and Porcupinefish

In this experiment, we prepared PSTs from toxic xanthid crab *Atergatis floridus* collected in Ishigaki Island, Okinawa Prefecture, Japan. The PST preparation comprised saxitoxin (STX), neoSTX, and decarbamoylSTX [20]. The PST content was determined using a high performance liquid chromatography (HPLC)-postcolumn detection method [26], assuming that each toxin of the STX group shows equal intensity/mol, and was collectively expressed as STXs.

**Figure 3:**
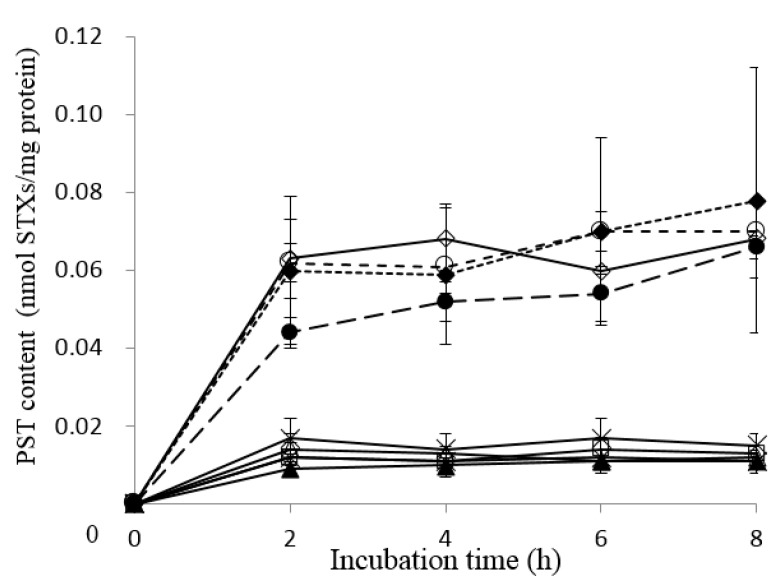
PST uptake into liver tissue slices of pufferfish, boxfish and porcupinefish. ◇ *Takifugu rubripes*; ● *Lagocephalus cheesemanii*; 〇 *Lagocephalus spadiceus*; ♦ *Sphoeroides pachygaster*; □ *Ostracion immaculatus*; ∆ *Diodon holocanthus*; ▲ *Diodon liturosus*; × *Diodon hystrix*; * *Chilomycterus reticulatus*.

**Figure 4:**
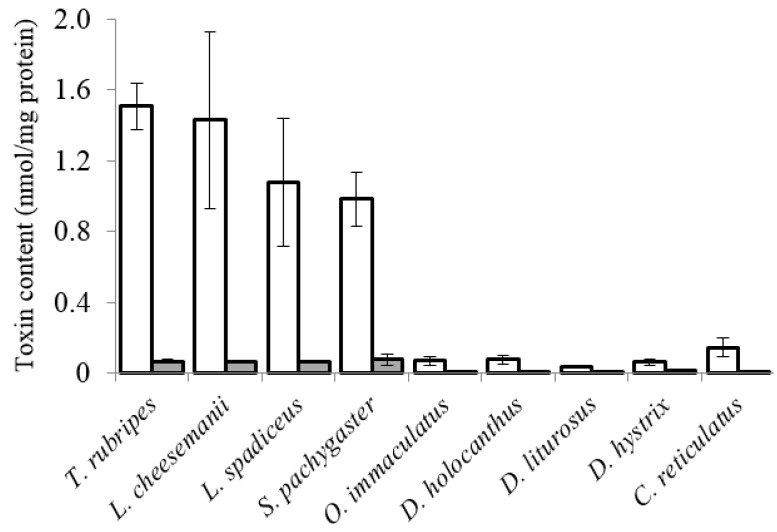
Comparison of the toxin content in liver tissue slices of pufferfish, boxfish and porcupinefish after 8 h incubation. 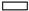 TTX; 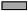 PSTs.

The profile of the PST content in liver tissue slices of the pufferfish differed from that of the boxfish and porcupinefish (Figure 3). Although the liver tissue slices of the pufferfish (*T. rubripes*, *L. spadiceus*, *L. cheesemanii* and *S. pachygaster*) contained 0.044–0.063 nmol STXs/mg protein after incubation for 2 h, the PST content did not increase with the incubation time up to 8 h. On the other hand, the contents of PST in the liver tissue slices of the boxfish and porcupinefish were below 0.02 nmol STXs/mg protein throughout the incubation time. The PST content in liver tissue slices from pufferfish, boxfish and porcupinefish, however, was significantly lower than the TTX content (*p* < 0.05; Figure 4). 

## 3. Discussion

This study revealed differences in the uptake of TTX and PSTs into liver tissue slices among four species of pufferfish in the family Tetraodontidae, one species of boxfish in the family Ostraciidae and four species of porcupinefish in the family Diodontidae by in vitro tissue culture. TTX was exclusively taken up into the liver tissue slices of pufferfish over time with incubation, while not into the liver tissue slices of boxfish or porcupinefish. Similarly, the PST content in the liver tissue slices of pufferfish was higher than that of boxfish and porcupinefish. However, the PST content was remarkably lower than the TTX content and did not increase over time with incubation, indicating that the liver of the fish examined in this study did not actively take up PST, irrespective of the fish species, compared with TTX. We previously reported that the liver of tiger pufferfish *T. rubripes* actively accumulates TTX [19,21,27] but not PST [20] based on in vitro tissue culture experiments. Therefore, these findings suggest that the pufferfish has a specific ability and function to take up TTX into the liver, like *T. rubripes* [21]. 

Notably, this is the first study to reveal that non-toxic pufferfish, *L. spadiceus*, *L. cheesemanii* and *S. pachygaster* also accumulated TTX in the liver when incubated with TTX and the TTX content in the liver tissue slices of the three species was comparable to that in *T. rubripes*. Wild specimens of *L. spadiceus* collected along Japanese coasts do not exhibit toxicity over 10 mouse unit (MU)/g where one MU is defined as the amount of toxin that kills a mouse in 30 min after intraperitoneal injection, which is the quarantine limit for the consumption of pufferfish in Japan [23,28,29]. The reason for the lack of toxicity in Japanese specimens of the three species of pufferfish is unclear. Here we discuss the possibilities that the three species of pufferfish do not accumulate TTX, as follows: ecologic habits of pufferfish and physiologic functions such as sensing mechanisms of TTX and resistance to TTX in pufferfish. 

It is thought that pufferfish are mainly toxified via the food chain, and therefore ecologic habits, including their feeding and habitation may affect pufferfish toxicity, although feeding habits of pufferfish remain unclear. A TTX-sensing mechanism in pufferfish may be associated with the toxification of pufferfish. Male pufferfish *T. niphobles* (at present *T. alboplumbeus*) is attracted to TTX, which plays a role as a sex pheromone at the time of spawning [30] and immature *T. rubripes* juveniles recognize TTX via their olfactory organ [31] to feed with TTX-containing marine organisms and accumulate TTX. In contrast, TTX-free fish are likely to avoid TTX. Itoi et al. [32] performed a predation experiment using non-toxic fish such as *Parablennius yatabei*, *Girella punctate* and *Chaenogobius annularis* as the predators, and grass pufferfish *T. niphobles* (at present *T*. *alboplumbeus*) larvae as the prey in small aquaria. Adult *Artemia* and medaka larvae were used as controls for the prey. When predators of non-toxic fish ingested the pufferfish larvae, they spat them out immediately, whereas they fed on *Artemia* and medaka. In addition, the gustatory organs of the rainbow trout *Oncorhynchus mykiss* and Arctic char *Salvelinus alpinus* respond sensitively to TTX at extremely low levels of 2 × 10^−7^ M for rainbow trout and of 1 × 10^−8^ M for Arctic char, despite the detection of L-proline, the most stimulatory amino acid for rainbow trout, at 1 × 10^−3^ M [33]. Moreover, pufferfish resistance to TTX may relate to the toxicity. Saito et al. [34] investigated the tolerance of pufferfish against intraperitoneally administered TTX. Toxic species of pufferfish, *T. rubripes*, *T. pardalis* and *T. niphobles* (at present *T. alboplumbeus*) exhibited higher resistance to TTX. They eventually died when 3000–7500 µg TTX per kilogram body weight was intraperitoneally injected. In contrast, *L. wheeleri* (at present *L. spadiceus*), *L. gloveri* (at present *L. cheesemanii*) and *S. pachygaster* exhibited markedly lower resistance to TTX administration, because they died by injection of only 150–200 µg TTX per kilogram body weight. Therefore, the three non-toxic species of pufferfish cannot accumulate a large amount of TTX, unlike the toxic species of pufferfish. It is most likely that pufferfish resistance to TTX administration is attributed to TTX-resistant skeletal muscle sodium channels. Pufferfish evolved resistance to TTX by amino acid substitutions of voltage-depend sodium channels [35,36], whereas the muscle fibers of the boxfish (*Rhynchostracion nasus*, at present *Ostracion nasus*) and the porcupinefish (*Diodon holocanthu*s) were sensitive to TTX [37]. Sodium channels of *L. spadiceus*, *L. cheesemanii* and *S. pachygaster* might be less tolerant against TTX than those of toxic species of pufferfish, although no reports have appeared concerning the protein structure of sodium channels in the three non-toxic pufferfish.

Furthermore, a specific plasma protein, pufferfish saxitoxin and tetrodotoxin-binding protein (PSTBP), may be implicated in the difference in toxification between toxic species and non-toxic species of pufferfish. PSTBP was first isolated from the plasma of *T. pardalis* as a toxin-binding protein [38]. The protein was detected in liver, intestine, ovary and skin of *T. pardalis* by immunohistochemical staining [39]. PSTBP is also found in the plasma of other toxic species of pufferfish in the genus *Takifugu* (*T. rubripes*, *T. poecilonotus* (at present *T. flavipterus*) [3], *T. snyderi*, *T. niphobles* (at present *T. alboplumbeus*) and *T. vermicularis*) as well as *T. pardalis*, but not in the plasma of TTX-free fish, such as the slime flounder *Microstomus achne*, rockfish *Sebastes schlegeli*, greenling *H. otakii* and Japanese flounder *Paralichthys olivaceus* [40]. Therefore, PSTBP is assumed to be a carrier protein that transfers TTX among tissues in toxic species of pufferfish in the genus *Takifugu*. Hashiguchi et al. [41] examined PSTBP genes in five toxic species of pufferfish in the genus *Takifugu* and three non-toxic species of pufferfish (*L. wheeleri* (at present *L. spadiceus*), *L. gloveri* (at present *L. cheesemanii*) and *S. pachygaster*). PSTBP genes were cloned in the genus *Takifugu*, while they were not obtained in the three non-toxic pufferfish. Thus, the non-toxic species of pufferfish without PSTBP could not be toxified with TTX, even if they orally consumed TTX through a TTX-containing diet. Any or all of the above-described mechanisms may contribute to the differences in toxification between toxic species and non-toxic species of pufferfish. Further studies with metagenomics analysis of prey organisms of the pufferfish, behavioral observation of the pufferfish using a Y-maze, rearing experiments of non-toxic species of pufferfish (*L. spadiceus*, *L. cheesemanii* and *S. pachygaster*) with TTX-containing feeds and protein analysis of sodium channels of the pufferfish will help to clarify the reasons for the differences. 

Finally, based on in vitro tissue culture, non-toxic species of pufferfish, *L. spadiceus*, *L. cheesemanii* and *S. pachygaster* potentially take up TTX in the liver with a toxification potency comparable to that of toxic species *T. rubripes* liver. Therefore, to prevent pufferfish poisoning, the liver of the three species of pufferfish should not be consumed. These findings are also important from the viewpoint of food safety. 

## 4. Materials and Methods 

### 4.1. Materials

Specimens of four species of pufferfish (*Takifugu rubripes*, *Lagocephalus spadiceus*, *Lagocephalus cheesemanii* (formerly *L. gloveri*) [20], and *Sphoeroides pachygaster* belonging to the family Tetraodontidae), one species of boxfish (*Ostracion immaculatus* belonging to the family Ostraciidae) and four species of porcupinefish (*Diodon holocanthus*, *Diodon liturosus*, *Diodon hystrix* and *Chilomycterus reticulatus* belonging to the family Diodontidae) were used. Specimens of *T. rubripes* were obtained from Tokyo central wholesale market (Tokyo, Japan). Specimens of *L. spadiceus*, *L. cheesemanii*, *S. pachygaster*, *O. immaculatus*, *D. holocanthus* and *C. reticulatus* were provided from Shimonoseki Marine Science Museum “Kaikyokan” (Yamaguchi, Japan), *D. liturosus* was obtained from the Blue Corner International Marine Aquarium Fish Supplier (Shizuoka, Japan) and *D. hystrix* was obtained from the Miyazu Energy Research Center Aquarium (Kyoto, Japan). All of specimens were transported live to the laboratory of Tokyo University of Marine Science and Technology and used for in vitro incubation experiments as described below.

TTX was purified from the ovaries of *T. pardalis* according to the previously reported method [19]. Briefly, TTX was extracted with acidified ethanol, defatted, ultrafiltered, treated with activated charcoal and chromatographed on a Bio-Gel P-2 column (Bio-Rad Laboratories, Hercules, CA, USA) and a Bio-Rex 70 column (Bio-Rad Laboratories), successively. PSTs were prepared from the toxic xanthid crab *Atergatis floridus* collected in Ishigaki Island, Okinawa Prefecture, Japan, by the method of Arakawa et al. [42]. Crystalline TTX was purchased from Wako Pure Chemical Industries (Osaka, Japan) and used as a standard for liquid chromatography tandem mass spectrometry analysis (LC-MS/MS). A certified standard of a mixture of neoSTX and decarbamoylSTX was donated by National Research Institute of Fisheries Science, Japan Fisheries Research and Education Agency, and used for HPLC analysis. All other chemicals were of analytical grade.

### 4.2. In Vitro Incubation Experiment of Liver Tissue Slices

Liver tissue slices of nine fish species were prepared according to the method described by Matsumoto et al. [21] with slight modifications. In brief, the liver was carefully excised from fish anesthetized by immersion into ice-cold seawater, and perfused through a portal vein with ice-cold perfusion buffer (160 mM NaCl, 5.4 mM KCl, 4.2 mM NaHCO_3_, 0.34 mM Na_2_HPO_4_, 0.44 mM KH_2_PO_4_, 10 mM HEPES and 5.6 mM D-glucose, adjusted to pH 7.4 with 2 M NaOH) using a handheld syringe. The liver was cut in slices 1-mm thick with microtome blades A22 (Feather Safety Razor, Osaka, Japan), and 8-mm diameter punches were obtained with a biopsy punch (Kai Industries, Gifu, Japan).

Each round slice was pre-incubated in transport buffer (160 mM NaCl, 4.8 mM KCl, 23.8 mM NaHCO_3_, 0.96 mM KH_2_PO_4_, 1.5 mM CaCl_2_, 1.2 mM MgSO_4_, 12.5 mM HEPES and 5.0 mM D-glucose, adjusted to pH 7.4 with 2 M NaOH) at 20 °C for 5 min with bubbling 95% O_2_–5% CO_2_ gas. The slices were placed into wells of a 24-well plate containing 1 mL of 50 µM TTX or 50 µM PSTs in the transport buffer (pH 7.4), and incubated at 20 °C for 8 h with bubbling 95% O_2_–5% CO_2_ gas. At 0, 2, 4, 6 and 8 h, the slices were removed from the incubation medium and rinsed with the ice-cold transport buffer. To each slice, 3 mL of 0.1% acetic acid was added, and the slices were homogenized with a BioMasher II (Asist, Tokyo, Japan), and ultrasonicated with an ultrasonic cleaner (2510J-MT, Bransonic, Danbury, CT, USA) for 5 min. Toxins and proteins were measured in the homogenates as described below.

In another series of experiment to examine the effect of the TTX concentration in the medium on TTX uptake into liver tissue slices of pufferfish *L. spadiceus*, the liver tissue slices were incubated with transport buffer containing 0–2000 µM TTX at 20 °C for 60 min. 

### 4.3. Determination of TTX and PSTs

TTX was extracted from the homogenates by heating for 10 min in a boiling water bath according to the standard method [43]. The toxin extracts were ultrafiltered through an Ultrafree-0.5 (nominal molecular weight cut-off 3000, Millipore, Billerica, MA, USA) and the resulting filtrates were used to analyze TTX. TTX was determined with a Waters Acquity UPLC and a TDQ triple-quadrupole tandem mass spectrometry (Waters, Milford, MA, USA). The analytical column was a TSKgel Amide-80 (2.0 × 150 mm, 3-µm particle size, Tosoh Corporation, Tokyo, Japan) for hydrophilic interaction liquid chromatography and maintained a 25 °C. The mobile phase consisted of 16 mM ammonium formate (pH 5.5)-acetonitrile (40:60, *v*/*v*) and was eluted at a flow rate of 0.2 mL/min. The eluate was induced into the ion source of electrospray ionization-mass spectrometry, ionized by the positive mode and detected in multiple reaction monitoring mode, *m*/*z* 320 > 162, with a collision energy of 45 eV.

As for PST extraction, aliquots of the homogenate were mixed with a tenth volume of 1 M HCl and heated for 5 min with a boiling water bath according to the standard method [44]. PSTs were separated by ion-pairing HPLC on a Mightysil RP-8 GP column (4.6 × 150 mm, 5-µm particle size, Kanto Chemical, Tokyo, Japan) with mobile phase of 10 mM ammonium phosphate buffer (pH 7.1) containing 4 mM sodium heptanesulfonic acid at a flow rate of 0.8 mL/min by the method of Oshima et al. [26]. PSTs were detected by a postcolumn oxidation reaction. The eluate was mixed with 70 mM periodic acid and 50 mM K_2_HPO_4_ in a reaction coil (φ0.5 mm × 10 m) at a total flow rate of 1.6 mL/min, at 65 °C. The toxins were monitored at 390 nm with 336 nm excitation using an Intelligent Fluorescence Detector FP-2020 Plus (Jasco, Tokyo, Japan). The PST preparation comprised mainly saxitoxins (STXs, data not shown). In this study, neoSTX and decarbamoylSTX were quantified using a standardized mixture of neoSTX (2.98 µM) and decarbamoylSTX (0.65 µM) provided by National Research Institute of Fisheries Science, Japan Fisheries Research and Education Agency. STX was determined by indirect quantification using decarbamoylSTX as an external standard to identify and quantify STX [45], because STX is listed as a Schedule 1 chemical warfare agent in the Chemical Weapons Convention, and production, stockpiling and utilization are strictly prohibited. 

### 4.4. Determination of Protein

The protein content was determined by the method of Lowry et al. [46] using bovine serum albumin as a standard. 

### 4.5. Statistical Analysis

The statistical significance of differences in the TTX and PST contents was evaluated using Student’s *t*-test. All data are presented as the mean ± standard deviation and *p* < 0.05 was considered significant.
